# Absorptive Capacity of Gingival Retraction Cords in Hemostatic Solutions: An In Vitro Study

**DOI:** 10.3390/medicina60081306

**Published:** 2024-08-13

**Authors:** Desislava R. Makakova, Plamen Zagorchev, Mariya Dimitrova, Yoanna Georgieva, Boris Tilov

**Affiliations:** 1 Department of Prosthetic Dentistry, Faculty of Dental Medicine, Medical University of Plovdiv, 4002 Plovdiv, Bulgaria; desislava.makakova@mu-plovdiv.bg (D.R.M.); yoanna.georgieva@mu-plovdiv.bg (Y.G.); 2 Department of Medical Physics, Biophysics and Mathematics, Faculty of Pharmacy, Medical University of Plovdiv, 4002 Plovdiv, Bulgaria; plamen.zagorchev@mu-plovdiv.bg; 3 Medical College, Medical University of Plovdiv, 4002 Plovdiv, Bulgaria; boris.tilov@mu-plovdiv.bg

**Keywords:** gingival retraction, pre-impression procedure, retraction cords, fixed prosthodontics, hemostatic solutions, mechanical–chemical retraction, absorption

## Abstract

*Background and Objectives:* Gingival retraction is a critical pre-impression procedure in fixed prosthodontics, crucial for exposing tooth margins and ensuring accurate impressions for restorations like crowns and bridges. This study aimed to evaluate the absorptive capacity of different gingival retraction cords. *Materials and Methods:* Ninety samples each of Ultrapak (Ultradent, South Jordan, UT, USA) #00, braided cord, coreless thread, and monofilament thread (totaling 270 samples) were immersed in 0.9% NaCl, 10% aluminum chloride, and 12.7% ferrous sulfate solutions for 120, 300, and 1200 s. The liquid absorption capacity was measured using a gravimetric method, and the data were analyzed using an F-test, setting the significance threshold at *p* < 0.05. *Results:* The results revealed statistically significant differences in absorption, particularly for aluminum chloride and ferric sulfate (*p* < 0.001). Ultrapak demonstrated the highest absorption, followed by the coreless cotton thread, while the monofilament thread absorbed the least, especially at 1200 s. *Conclusions:* These findings indicate that Ultrapak’s superior absorption could enhance moisture control during procedures, highlighting the importance of selecting an appropriate retraction cord for optimal clinical outcomes. Further research is needed to confirm these findings in a clinical setting.

## 1. Introduction

The process of taking an impression of a fixed-type prosthesis involves several key steps: displacement of the gingival tissue, capturing an impression of the prosthetic field (which includes the prepared tooth, part or all of the dentition, and adjacent tissues), and recording the antagonist’s teeth [1,2]. The initial step, gingival retraction or displacement, commonly involves using a mechanical device, such as a string or paste, placed in the gingival sulcus [3,4]. The objective of gingival retraction is to create a space of approximately 0.2 mm in the periodontal sulcus [5,6]. This gap is essential for controlling bleeding and minimizing gingival fluid secretion [7]. It ensures clear exposure of the tooth preparation margins, which is crucial for achieving accurate impressions and a precise prosthetic fit. Effective gingival retraction not only improves impression accuracy but also contributes to the overall success of prosthetic restoration by ensuring optimal fit, function, and aesthetics [8]. Various techniques and materials are available for gingival retraction, including braided or knitted cords, retraction pastes, and gels, which are often impregnated with hemostatic agents, such as aluminum chloride or ferric sulfate. These agents help control bleeding and reduce crevicular fluid flow, thereby maintaining a dry field for impression-taking [9]. The choice of retraction technique and material can significantly influence the clinical outcome. Factors such as the degree of gingival inflammation, depth of the sulcus, and specific characteristics of tooth preparation must be considered [10]. Additionally, proper application techniques and gentle handling of gingival tissues are crucial to prevent trauma and ensure effective retraction [11].

The techniques for gingival retraction can be classified into three primary categories: mechanical, mechano-chemical, and surgical [12]. A combination of these methods is typically used, with mechano-chemical being the most common. The choice of retraction technique depends on the clinical situation and the clinician’s preferences [13,14]. Mechanical methods involve physically displacing the gingiva using tools such as retraction cords, rings, or packing instruments [15]. These methods are often simple and effective for minor gingival displacement.

Mechano-chemical methods for gingival retraction combine mechanical displacement with chemical agents to enhance the effectiveness of the procedure [16,17]. The chemical agents used in these methods are classified into two categories: class 1 includes vasoconstrictors like racemic epinephrine (8%), and class 2 encompasses hemostatic and astringent agents such as aluminum chloride, iron trisulfate, aluminum sulfate, alum, and zinc chloride [18,19]. These agents help to control bleeding and reduce crevicular fluid flow, thereby maintaining a dry field for accurate impression-taking [20]. Surgical methods, such as electrosurgery and laser surgery, are less common but are employed when significant gingival retraction is needed to provide clear access to tooth margins [21,22].

The choice of retraction method in clinical practice depends on factors like the extent of gingival overgrowth, inflammation level, sulcus depth, and the specific requirements of the dental procedure [23]. The clinician’s experience and familiarity with different techniques also play a significant role. The mechanical–chemical approach is often preferred because of its effectiveness in achieving both retraction and hemostasis, which ensures optimal exposure of tooth margins for accurate impressions and enhances the success of prosthetic restorations [24,25].

Advancements in retraction materials and techniques aim to improve patient comfort, reduce tissue trauma, and achieve more predictable clinical outcomes [26,27,28]. Ongoing research focuses on refining existing methods and exploring new approaches to enhance the efficiency and effectiveness of gingival retraction [29]. Hemostatic solutions used for retraction must be effective, safe, reversible after removal, and quickly removable without causing permanent tissue displacement [30]. Proper saturation of retraction cords with these agents ensures sufficient tissue retraction, creating sufficient space for impression materials to accurately capture preparation margins and be removed without tearing [31,32].

Meeting these conditions is essential for obtaining precise dental impressions [33]. Effective retraction and control of gingival tissues allow for accurate recording of tooth margins, which is crucial for fabricating well-fitting prosthetic restorations [34]. Moreover, the ability to remove retraction materials without leaving residue or causing lasting tissue displacement helps maintain gingival health and integrity. The inclusion of hemostatic agents in retraction cords also helps manage bleeding and reduce crevicular fluid flow, which is vital for establishing a dry operative field during impression-taking, thereby improving both impression accuracy and the overall effectiveness of prosthetic treatments [35,36].

Intraoral scanning has transformed gingival displacement techniques by enabling direct digital capture of the oral cavity, thus eliminating the need for traditional impression materials [4]. This technology not only improves patient comfort but also minimizes the risk of distortion that can occur with conventional impressions, offering high-precision, real-time imaging of the gingival sulcus and adjacent areas [12]. Additionally, using desktop scanners to create digital models from physical casts after an initial impression provides a valuable method for integrating traditional impressions into digital workflows. This combination of methods enhances the accuracy of final restorations and streamlines communication among dental professionals. Furthermore, the use of desktop scanners to digitize physical casts obtained from conventional impressions offers a bridge between traditional and digital methodologies. This hybrid approach enables the creation of detailed digital models from traditional impressions, facilitating improved accuracy in restorations and smoother integration of digital technologies into existing workflows [25].

In the realm of dental prosthodontics, the selection of retraction cords and retraction solutions plays a crucial role in the success of impression techniques. Previous studies have extensively examined how different types and materials of retraction cords affect their performance. For instance, one research demonstrated that the type of retraction cord can significantly influence fluid absorption and gingival displacement, emphasizing the need for careful selection based on clinical requirements [14]. Similarly, another study highlighted the varying fluid absorption capacities of retraction cords made from different materials, including coreless braided cotton and those with polyamide cores [9]. Their findings revealed that cords made from alum-treated materials exhibited superior absorption compared to those soaked in other solutions.

In addition to the cord types and materials, the choice of retraction solution has also been a focus of investigation. Studies have shown that the efficacy of retraction solutions, such as ferric sulfate and aluminum chloride, can significantly impact the performance of retraction cords. For example, one research examined the absorbency of retraction cords soaked in various astringent solutions and found notable differences in fluid uptake and gingival displacement based on the type of solution used [16]. Their work underscores the importance of selecting an appropriate retraction solution to optimize the effectiveness of retraction cords and achieve precise impressions.

Ongoing advancements in gingival retraction have aimed to improve the efficacy, safety, and comfort of retraction materials and techniques. However, there is a notable scientific gap in the understanding of the comparative fluid absorption capacities of different retraction cords used in fixed prosthodontics. This study addresses this gap by evaluating and comparing the fluid absorption capabilities of various retraction cords. This research provides valuable data on how these cords perform with hemostatic solutions, which is crucial for achieving effective retraction and maintaining a dry field for accurate impression-taking. The findings will help clinicians select the most effective retraction materials, enhance the fit, function, and aesthetics of prosthetic restorations, and improve overall patient outcomes.

## 2. Materials and Methods

### 2.1. Selection of Retraction Cords

This study investigated the fluid absorption properties of three types of retraction cords, as presented in Table 1.

**Figure 1:**
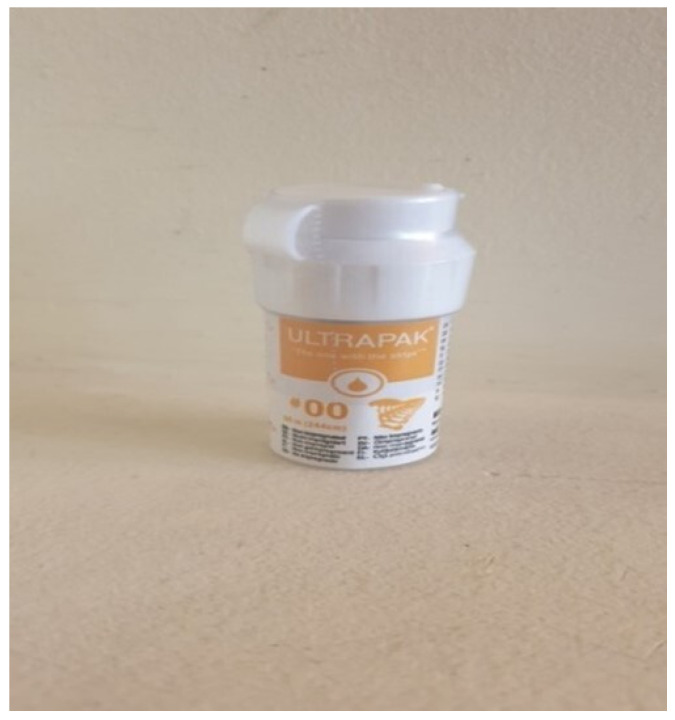
Ultrapak #00 (Ultradent, UT, USA) retraction cord (Group U).

**Figure 2:**
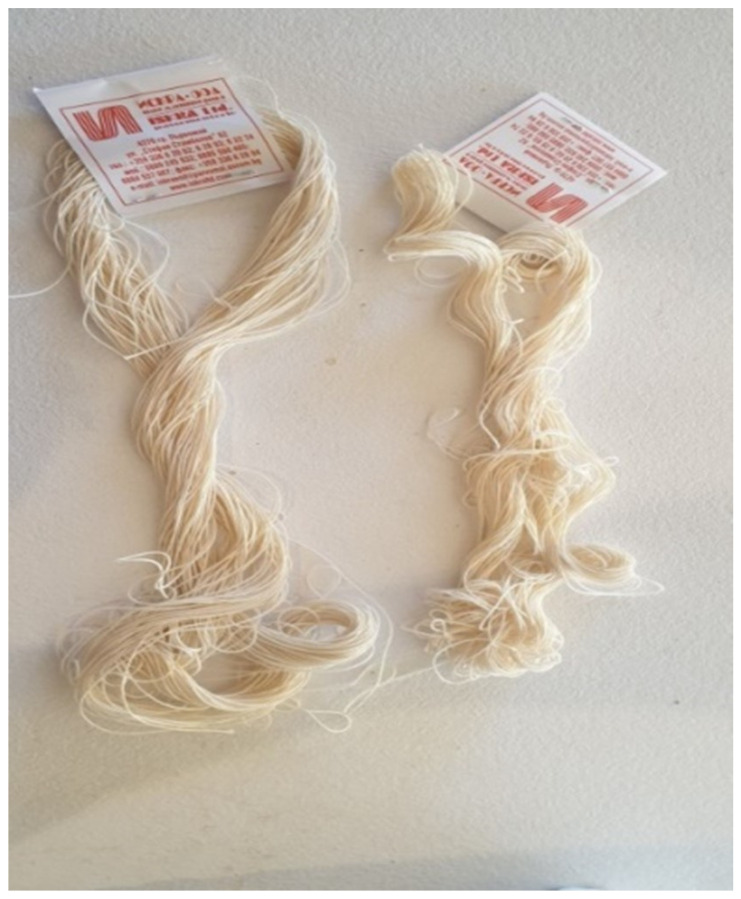
Braided cords (Groups P and PP) (Iskra Ltd., Parvomay, Bulgaria).

The cords were selected to compare their performance in fixed prosthodontics and assess their potential clinical advantages (Figure 1 and Figure 2).

### 2.2. Sample Size and Preparation

A total of 270 samples were prepared, with 90 samples from each type of cord. Each sample was standardized to a length of 50 mm. The sample size was determined based on preliminary trials and statistical power calculations to ensure adequate power for detecting significant differences in fluid absorption [37]. Sample size calculations were performed using G*Power software (Version 3.1.9.7, Düsseldorf University, Düsseldorf, Germany). The samples were divided into three groups of 30 per cord type based on the immersion solution used.

### 2.3. Hemostatic Solutions

**Figure 3:**
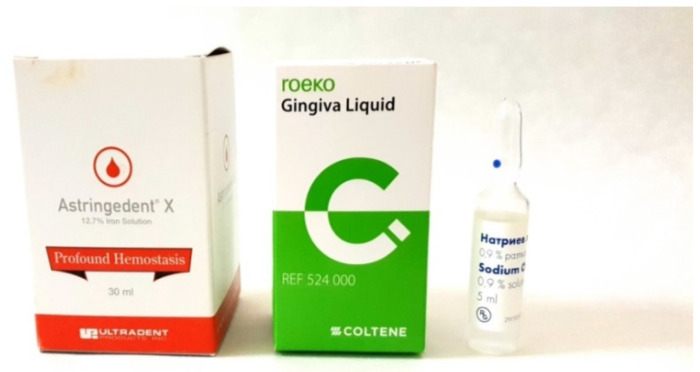
Hemostatic solutions administered as medication to retract the gingival sulcus.

The solutions for gingival sulcus retraction included the following:

- 0.9% NaCl
- 10% aluminum chloride (Al_2_Cl_3_) by Roeko (Coltene, Maribor, Slovenia)
- 12.7% ferric sulfate (Fe_2_(SO_4_)_3_) by Ultradent (Ultradent, South Jordan, UT, USA) (Figure 3)

### 2.4. Experimental Procedure

**Figure 4:**
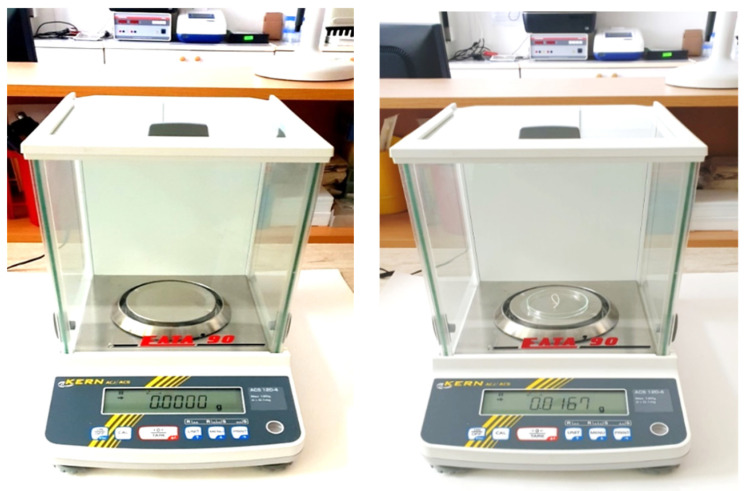
Measurement of the mass of the used cord together with the absorbed fluid by analytical balance ACS 120-4 (Kern, Lohmar, Germany).

To ensure the accuracy and reliability of the measurements, the retraction cords were weighed three times by different researchers before and after incubation, and all data were meticulously documented. Each group of 30 samples per cord type was immersed in 50 mL glass beakers containing one of the hemostatic solutions at three different intervals: 120 s, 300 s, and 1200 s. These intervals were chosen to simulate varying clinical conditions and to assess how time affects fluid absorption. After immersion, the excess liquid on the samples was removed using a qualitative analysis filter paper soaked in the respective solution (Figure 4). Immediately before immersion, any air pockets that could have impeded the internal absorption of moisture were carefully expelled by manual compression.

### 2.5. Measurement and Data Analysis

The fluid absorption capacity was calculated by weighing each sample before and after immersion three times using an analytical balance (ACS 120-4, Kern, Lohmar, Germany) with a precision of ±0.1 mg. The measurements were conducted independently by three researchers to ensure accuracy and reliability. The weight difference indicated the amount of liquid absorbed. Statistical analysis was performed using an F-test (IBM, SPSS Statistics, Version 2023, IBM Corp., Chicago, IL, USA) to compare the absorption capacities across different cord types and immersion times, with the significance threshold set at *p* < 0.05. This analysis aimed to identify significant differences and determine the performance of each retraction cord.

### 2.6. Study Design

This in vitro study aimed to assess the fluid absorption characteristics of different retraction cords used in fixed prosthodontics. The design was structured to compare the performance of the three types of retraction cords under controlled laboratory conditions. This approach aligns with established methodologies and ensures the accurate measurement of absorption under different conditions [37,38,39]. This study evaluated the absorption capacity of each cord type when exposed to various hemostatic solutions and immersion times, simulating typical clinical scenarios.

## 3. Results

The results showed the maximum amounts of fluid absorbed by the different retraction cords in 0.9% NaCl, 12.7% Fe_2_(SO_4_)_3_, and 10% Al_2_Cl_3_ solutions, as presented in Table 2.

The comparison of retraction cords immersed in NaCl across different groups and time intervals revealed significant variations. In the 120-s group, a comparison between groups P and U showed a substantial difference (*p* < 0.001), 95% CI: −0.6630) to (−0.2704), as did the comparison between groups PP and U (*p* < 0.001), 95% CI: (−0.4996) to (−0.1070). However, the difference between groups P and PP was not statistically significant (*p* > 0.05). Similar trends were observed in the 300-s and 1200-s groups, where the differences between groups P and U, as well as PP and U, were statistically significant (*p* < 0.001). In contrast, the differences between groups P and PP were not significant (*p* > 0.05). These results suggest that the retraction cords in group U differ significantly from those in groups P and PP across all time intervals. In contrast, the differences between groups P and PP were minimal and not statistically significant.

Statistically significant differences were observed in the study evaluating retraction cords immersed in an aluminum chloride solution. Specifically, significant differences were found in comparisons between group U vs. group P cords, group U vs. group PP cords, and group P vs. group PP cords. The most pronounced statistical difference was noted between the group U and group PP cords at 1200 s, with a t-value of 22.26 (*p* < 0.001), at 300 s with a t-value of 18.64 (*p* < 0.001), and at 120 s with a t-value of 22.26 (*p* < 0.001) (Table 1).

A noticeable difference was found between the U and P cord groups, with t-values of 12.11 (*p* < 0.001) at 300 s, 11.18 (*p* < 0.001) at 120 s, and 11.01 (*p* < 0.001) at 1200 s. Similarly, significant differences were identified between the P and PP cord groups, with t-values of 11.25 (*p* < 0.001) at 1200 s, 7.86 (*p* < 0.001) at 300 s, and 6.76 (*p* < 0.001) at 120 s (Table 1).

These results highlight the significant variations in the fluid absorption capacities of different retraction cords when immersed in aluminum chloride solutions for various time intervals. This underscores the importance of selecting the appropriate cord type in clinical practice, as it can significantly impact the effectiveness and outcomes of gingival retraction procedures in fixed prosthodontics.

When retraction cords were tested in a 12.7% ferric sulfate solution, significant statistical differences were also found between the PP and U cord groups at different times: t = 6.15 (*p* < 0.001) at 120 s, t = 10.42 (*p* < 0.001) at 300 s, and t = 11.03 (*p* < 0.001) at 1200 s. A similar pattern emerged in the comparison between the P and PP cord groups, with significant differences at 1200 s (t = 6.39, *p* < 0.001), 300 s (t = 6.39, *p* < 0.001), and 120 s (t = 5.97, *p* < 0.001) (Table 1).

When evaluating the cords in a Fe_2_(SO_4_)_3_ solution, no statistically significant difference was found between group U and group P cords at 120 s (*p* > 0.05) and 300 s (*p* > 0.05), but a significant difference was noted at 1200 s, with t = 4.63 (*p* < 0.001) (Table 1).

Similarly, when examining retraction cords immersed in a NaCl solution, significant statistical differences were observed between group P and group U cords at 1200 s, t = 13.69 (*p* < 0.001); at 300 s, t = 12.49 (*p* < 0.001); and at 120 s, t = 8.789 (*p* < 0.001). A comparable trend was observed between group PP and group U cords, with significant differences at 1200 s, t = 11.43 (*p* < 0.001); at 300 s, t = 11.43 (*p* < 0.001); and at 120 s, t = 5.71 (*p* < 0.001). In contrast, no statistically significant difference (*p* > 0.05) was found between group P and group PP cords when tested separately across various time intervals (Table 1).

A comparison of the effects of NaCl, Al2Cl3, and Fe_2_(SO_4_)_3_ on retraction cords across different time intervals (120 s, 300 s, 1200 s) reveals distinct trends in how each solution influences the properties of the cords over time.

- 120 s Interval:

In the NaCl solution, the comparison between groups P and PP showed no significant differences (*p* > 0.05). However, significant differences were observed between groups P and U (*p* < 0.001) and between groups PP and U (*p* < 0.001), indicating that both treated groups (P and PP) differed substantially from the untreated group (U).

For Al_2_Cl_3_, all comparisons revealed significant differences (*p* < 0.001), demonstrating that this solution caused marked changes in retraction cord properties across all group comparisons (P vs. PP, P vs. U, and PP vs. U).

**Figure 5:**
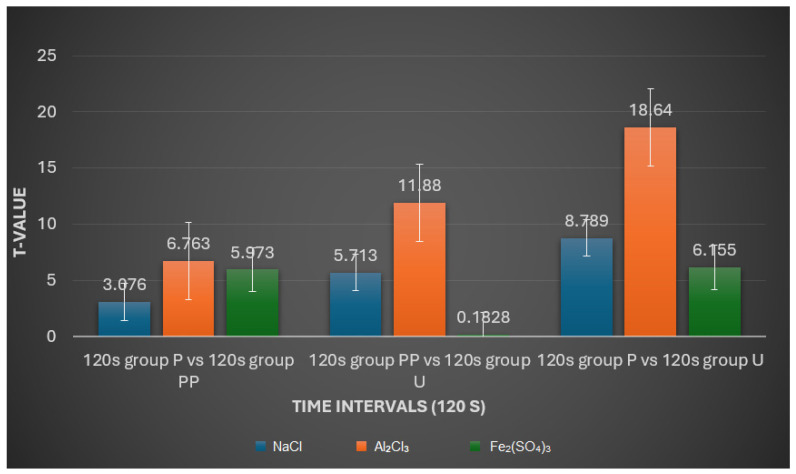
An interactive plot comparing t-values with time interval (120 s).

In the Fe_2_(SO_4_)_3_ solution, significant differences were observed between groups P and PP (*p* < 0.001) and between groups PP and U (*p* < 0.001), but not between groups P and U (*p* > 0.05). This suggests that Fe_2_(SO_4_)_3_ significantly alters the properties of the cords in group PP compared to the other two groups within the 120 s interval (Figure 5).

- 300 s Interval:

At the 300 s interval in NaCl, the results were similar to the 120 s interval, with no significant difference between groups P and PP (*p* > 0.05) but significant differences between groups P and U (*p* < 0.001) and between groups PP and U (*p* < 0.001).

For Al_2_Cl_3_, significant differences across all comparisons (P vs. PP, P vs. U, and PP vs. U) persisted (*p* < 0.001), indicating that the pronounced effects of this solution continue over time.

**Figure 6:**
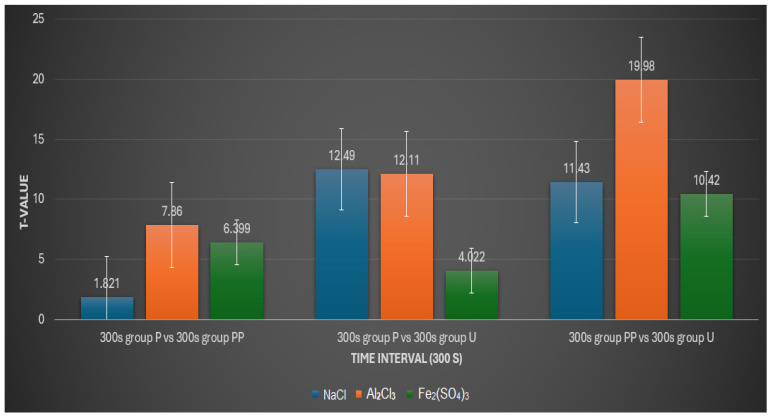
An interactive plot comparing t-values with time interval (300 s).

In the Fe_2_(SO_4_)_3_ solution, all comparisons showed significant differences (*p* < 0.05), with P vs. PP and PP vs. U showing highly significant differences (*p* < 0.001). This indicates that by 300 s, even the comparison between groups P and U showed significant changes, albeit to a lesser degree compared to the other two comparisons (Figure 6).

- 1200 s Interval:

After 1200 s in NaCl, the pattern remains consistent with the earlier intervals. No significant difference is observed between groups P and PP (*p* > 0.05), while significant differences persist between groups P and U (*p* < 0.001) and PP and U (*p* < 0.001).

For Al_2_Cl_3_, the trend of significant differences across all group comparisons (*p* < 0.001) continues, indicating the sustained and strong impact of this solution on retraction cord properties.

**Figure 7:**
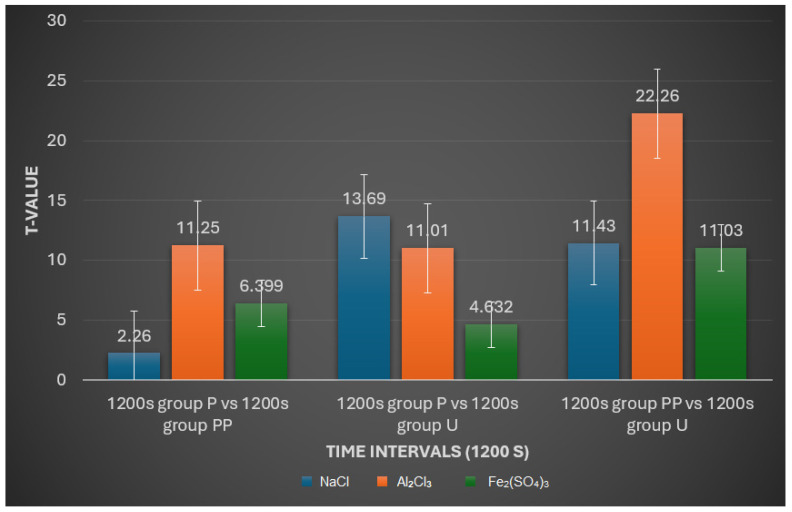
An interactive plot comparing t-values with time interval (1200 s).

In the Fe_2_(SO_4_)_3_ solution, all group comparisons show significant differences (*p* < 0.001), demonstrating that the substantial effects of this solution on retraction cord properties are evident across all groups at 1200 s intervals (Figure 7).

Across all time intervals, NaCl consistently shows significant differences between the untreated group (U) and the treated groups (P and PP), with no significant difference between P and PP. Al_2_Cl_3_ produces significant differences in all pairwise comparisons across all time intervals, indicating the most pronounced and consistent changes in retraction cord properties. Fe_2_(SO_4_)_3_ shows significant differences primarily involving PP, with delayed significant effects between P and U becoming apparent after 120 s.

In conclusion, Al_2_Cl_3_ exhibits the most consistent and significant differences across all comparisons and time intervals, indicating a strong and sustained effect on retraction cord properties. NaCl primarily shows significant differences between the untreated and treated groups, while Fe_2_(SO_4_)_3_ shows significant differences mainly involving the PP group, with delayed effects between P and U.

## 4. Discussion

Gingival retraction is a crucial procedure for obtaining accurate impressions of fixed prosthetic restorations, particularly for preparation margins positioned at or below the level of the gingival margin. The primary technique for achieving displacement involves the use of a retraction cord soaked in a medicated solution. This study aimed to evaluate and compare the fluid absorption capacities of different types of retraction cords commonly employed in fixed prosthodontics. Specifically, this study focused on assessing how well these cords absorb solutions commonly used for gingival retraction, such as 0.9% NaCl, 12.7% Fe_2_(SO_4_)_3_, and 10% Al_2_Cl_3_. These solutions are critical for creating a dry and clear field around the tooth preparation, which is essential for obtaining precise impressions. The findings highlight substantial variations in fluid absorption capacities among different types of retraction cords when exposed to Fe_2_(SO_4_)_3_ and NaCl solutions for varying durations. These results underscore the importance of selecting retraction cords based on their specific performance characteristics, as these properties can significantly influence the effectiveness of gingival retraction procedures in clinical practice.

In a study by Patel et al., it was established that the optimal soaking duration for maximizing fluid absorption by retraction cords is approximately 20 min [40]. This finding is corroborated by the current study, which also observed that both the Ultrapak cord and the newly developed threads achieved peak absorbency within this 20 min period [41]. This consistency underscores the reliability of a 20 min soaking duration for enhancing the performance of retraction cords. In clinical practice, immersing retraction cords in astringent solutions for 20 min is crucial for effective gingival displacement. This duration allows the cords to become thoroughly saturated, which helps efficiently move the gingival tissues away from the preparation area. Consequently, this creates the necessary space for impression materials to accurately capture and delineate preparation margins. Such preparation is essential for achieving a precise and comprehensive impression, which directly impacts the success of fixed prosthodontic restorations [42].

Additionally, adequate soaking time is vital for preserving the integrity of the impression. Properly soaked retraction cords can be removed from the sulcus more easily and cleanly without disturbing the impression material or compromising its accuracy. This is important for maintaining the quality of the impression, as any displacement or distortion can lead to inaccuracies in the final prosthetic restoration. The alignment of results between this study and Patel et al.’s research highlights the robustness of the 20 min soaking duration as a standard practice. This standardization is advantageous for ensuring consistency in clinical procedures and outcomes. Adhering to this well-established soaking time allows clinicians to optimize the performance of retraction cords, thereby enhancing the overall effectiveness of gingival retraction techniques.

The selected time intervals in this study are consistent with those employed in analogous research endeavors conducted by other researchers [37,40,42]. This methodological alignment ensures consistency across studies and facilitates meaningful comparisons of results within the field of dental impression procedures. By adhering to standardized time intervals for soaking retraction cords in medicated solutions, researchers can reliably evaluate and optimize their performance characteristics. This approach not only enhances the scientific rigor of the investigations but also supports the practical application of findings in clinical settings [43]. It underscores the pivotal role of timing in achieving optimal tissue retraction and fluid absorption, which is crucial for obtaining accurate dental impressions necessary for successful prosthetic restorations.

Furthermore, these findings emphasize the importance of methodological standardization in advancing dental research and improving the efficacy of retraction techniques [44]. Consistent protocols enable clinicians to make informed decisions regarding the selection and application of retraction cords, thereby enhancing their overall reliability and effectiveness in improving patient care outcomes. In their study, Kansal et al. investigated the fluid absorption characteristics of retraction cord samples soaked for 20 min [45]. Their results revealed that cords immersed in alum solution exhibited the highest liquid absorption, surpassing those soaked in 21% ferric sulfate solution. This finding aligns with that of the current study, reinforcing the notion that soaking duration is crucial for optimizing fluid uptake in retraction cords. Kansal et al.‘s work emphasizes the effectiveness of alum solutions in achieving superior absorption, which may be attributable to the chemical properties of alum that enhance fluid interaction with the cord material.

Moreover, Kansal et al. employed a standardized soaking time of 10 min for plasma and artificial saliva solutions across all their samples, recommending this duration for optimal tissue retraction and fluid absorption within the gingival sulcus [37,46]. This shorter soaking time reflects a different approach compared to the 20 min soaking duration, yet it highlights the importance of the duration in achieving effective gingival retraction. The use of plasma and artificial saliva solutions, which likely have distinct chemical and physical properties compared to alum and ferric sulfate, suggests that different soaking times may be optimal, depending on the type of solution used and the specific retraction cord material used.

The contrast between Kansal et al.’s findings with alum and ferric sulfate and the current study’s observations, alongside the use of a 10 min soaking period for plasma and artificial saliva, underscores the complexity of fluid absorption dynamics in retraction cords. The varying results based on soaking solutions and durations point to the need for tailored soaking protocols depending on the specific clinical scenario and properties of the retraction cords and solutions used. For instance, although alum solutions may offer superior absorption in certain contexts, other solutions may require different soaking durations to achieve comparable results. The choice of a 10 min soaking period is supported by earlier studies conducted by Kumbuloglu, Hao, Kazakova, and Nowakowska, all of which recommend 5–10 min for retraction cords to effectively displace gingival tissue [47,48,49,50]. This established timeframe has been shown to consistently achieve optimal results in preparing gingival tissue for dental impressions. By adhering to this duration, clinicians can ensure a standardized approach that enhances the reliability and effectiveness of the impression-taking process. Such methodological rigor is crucial in clinical practice to maintain uniformity and quality in dental procedures, thereby supporting accurate treatment outcomes and patient satisfaction [51,52,53].

Comparatively, while Patel et al. and Kansal et al. highlight the benefits of longer soaking durations (20 min), other studies suggest that shorter periods can also be effective. This discrepancy indicates the potential for tailored approaches depending on the specific clinical situations and materials used. Ultimately, these findings underscore the necessity for ongoing research to refine soaking protocols, ensuring that they are both effective and practical for diverse clinical scenarios.

This study has several limitations that warrant consideration. Firstly, the sample size and diversity of retraction cords tested may limit the generalizability of the findings. A larger and more diverse sample pool, including pre-impregnated retraction cords, would provide a broader perspective on how different types of cords perform across various clinical contexts and patient demographics. Secondly, the study’s reliance on laboratory simulations rather than clinical settings could affect the translation of results to real-world applications. Future research should validate these findings in clinical settings to ensure their applicability and reliability in dental practice. Thirdly, the study focused on short-term fluid absorption within specific time intervals, overlooking potential long-term effects and durability of the cords under repeated use. Additionally, using fixed concentrations of retraction solutions limits insights into how varying solution strengths or combinations might influence cord performance. Lastly, while the gravimetric method used to measure fluid absorption is standard, it may not fully capture all aspects of cord performance relevant to clinical efficacy, such as tissue response and handling characteristics.

Future research should consider conducting clinical trials to assess retraction cord performance across diverse patient populations under varying clinical conditions. Longitudinal studies would be beneficial in determining the durability and sustained effectiveness of retraction cords over extended periods of use. Comparative studies are particularly important for evaluating the efficacy of different cord materials, designs, and soaking protocols, including a direct comparison with pre-impregnated retraction cords. Such comparisons can help determine whether pre-impregnated cords offer superior or comparable performance to traditional cords in terms of fluid absorption, tissue management, and ease of use. Collaborative multicenter research efforts could enhance sample diversity and validate findings across different geographical and practice settings, providing a more comprehensive understanding of cord performance. Additionally, integrating biomechanical and histological analyses would offer deeper insights into the biological effects of retraction cords on gingival tissues, potentially revealing differences in tissue responses and healing outcomes. Addressing these limitations and pursuing future research avenues will contribute to refining our understanding of retraction cord performance and ultimately improve clinical outcomes in fixed prosthodontics.

## 5. Conclusions

In this study, it was observed that the optimal duration of fluid absorption was 1200 s. During this time, the Ultrapak cord demonstrated superior absorption compared to the developed coreless cotton thread and monofilament thread. The amount of fluid absorbed is influenced by factors such as immersion time, solution type, and specific characteristics of the retraction cord, all of which play crucial roles in achieving successful gingival retraction for dental impressions. These findings underscore the importance of selecting appropriate retraction materials and optimizing procedural timing to enhance the efficacy and reliability of retraction techniques in clinical practice. Future research could further explore how varying these parameters impacts overall treatment outcomes and patient care in fixed prosthodontics.

## Figures and Tables

**Table 1:** Types of materials used in this study.

Name	Composition	Braiding Method	Manufacturer
Ultrapak #00 (Group U)	Cotton	Knitted	Ultradent Products, South Jordan, UT, USA
Coreless Braided Cotton Cords (Group P)	Cotton	Coreless, Braided	Iskra Ltd., Parvomay, Bulgaria
Braided Cords with a Polyamide Core (Group PP)	Cotton with Polyamide	Braided with Polyamide Monofilament Core	Iskra Ltd., Parvomay, Bulgaria

**Table 2:** Bonferroni’s Multiple Comparison Test—Absorption (Abs fluid g/g cord) of the cords (groups U, P, PP) for the three solutions (NaCl, Al_2_Cl_3_, Fe_2_(SO_4_)_3_) and a soaking time of 1200 s.

Bonferroni’s Multiple Comparison Test	Mean Difference	*t*	*p*	95% CI of Difference
**NaCl**				
120 s group P vs. 120 s group PP	−0.1633	3.076	*p* > 0.05	−0.3596 to 0.03296
120 s group P vs. 120 s group U	−0.4667	8789	*p* < 0.001	−0.6630 to −0.2704
120 s group PP vs. 120 s group U	−0.3033	5.713	*p* < 0.001	−0.4996 to −0.1070
300 s group P vs. 300 s group PP	−0.09667	1.821	*p* > 0.05	−0.2930 to 0.09962
300 s group P vs. 300 s group U	−0.6633	12.49	*p* < 0.001	−0.8596 to −0.4670
300 s group PP vs. 300 s group U	−0.5667	11.43	*p* < 0.001	−0.7630 to −0.3704
1200 s group P vs. 1200 s group PP	−0.1200	2.260	*p* > 0.05	−0.3163 to 0.07629
1200 s group P vs. 1200 s group U	−0.7267	13.69	*p* < 0.001	−0.9230 to −0.5304
1200 s group PP vs. 1200 s group U	−0.6067	11.43	*p* < 0.001	−0.8030 to −0.4104
**Al_2_Cl_3_**				
120 s group P vs. 120 s group PP	0.2867	6.763	*p* < 0.001	0.1300 to 0.4434
120 s group P vs. 120 s group U	−0.5033	11.88	*p* < 0.001	−0.6600 to −0.3466
120 s group PP vs. 120 s group U	−0.7900	18.64	*p* < 0.001	−0.9467 to −0.6333
300 s group P vs. 300 s group PP	0.3333	7864	*p* < 0.001	0.1766 to 0.4900
300 s group P vs. 300 s group U	−0.5133	12.11	*p* < 0.001	−0.6700 to −0.3566
300 s group PP vs. 300 s group U	−0.8467	19.98	*p* < 0.001	−1.003 to −0.6900
1200 s group P vs. 1200 s group PP	0.4767	11.25	*p* < 0.001	0.3200 to 0.6334
1200 s group P vs. 1200 s group U	−0.4667	11.01	*p* < 0.001	−0.6234 to −0.3100
1200 s group PP vs. 1200 s group U	−0.9433	22.26	*p* < 0.001	−1.100 to −0.7866
**Fe_2_(SO_4_)_3_**				
120 s group P vs. 120 s group PP	0.3267	5973	*p* < 0.001	0.1245 to 0.5289
120 s group P vs. 120 s group U	−0.01000	0.1828	*p* > 0.05	−0.2122 to 0.1922
120 s group PP vs. 120 s group U	−3.3367	6.155	*p* < 0.001	−0.5389 to −0.1345
300 s group P vs. 300 s group PP	0.3500	6.399	*p* < 0.001	0.1478 to 0.5522
300 s group P vs. 300 s group U	−0.2200	4.022	*p* < 0.05	−0.4222 to −0.01781
300 s group PP vs. 300 s group U	−0.5700	10.42	*p* < 0.001	−0.7722 to −0.3678
1200 s group P vs. 1200 s group PP	0.3500	6.399	*p* < 0.001	0.1478 to 0.5522
1200 s group P vs. 1200 s group U	−0.2533	4.632	*p* < 0.001	−0.4555 to −0.05114
1200 s group PP vs. 1200 s group U	−0.6033	11.03	*p* < 0.001	−0.8055 to −0.4011

## Data Availability

Data are contained within the article.
